# Pseudotargeted Metabolomic Fingerprinting and Deep Learning for Identification and Visualization of Common Pathogens

**DOI:** 10.3389/fmicb.2022.830832

**Published:** 2022-03-10

**Authors:** Ying Feng, Moutong Chen, Xianhu Wei, Honghui Zhu, Jumei Zhang, Youxiong Zhang, Liang Xue, Lanyan Huang, Guoyang Chen, Minling Chen, Yu Ding, Qingping Wu

**Affiliations:** ^1^Guangzhou Institute of Chemistry, Chinese Academy of Sciences, Guangzhou, China; ^2^Guangdong Provincial Key Laboratory of Microbial Safety and Health, Ministry of Agricultural and Rural Affairs, Key Laboratory of Agricultural Microbiomics and Precision Application, State Key Laboratory of Applied Microbiology Southern China, Guangdong Institute of Microbiology, Guangdong Academy of Sciences, Guangzhou, China; ^3^University of Chinese Academy of Sciences, Beijing, China; ^4^Department of Food Science and Technology, Institute of Food Safety and Nutrition, Jinan University, Guangzhou, China

**Keywords:** pseudotargeted metabolomic, deep learning, LC–QQQ–MS, variational autoencoder (VAE), convolutional neural network (CNN)

## Abstract

Matrix-assisted laser desorption/ionization time-of-flight mass (MALDI-TOF) spectrometry fingerprinting has reduced turnaround times, costs, and labor as conventional procedures in various laboratories. However, some species strains with high genetic correlation have not been directly distinguished using conventional standard procedures. Metabolomes can identify these strains by amplifying the minor differences because they are directly related to the phenotype. The pseudotargeted metabolomics method has the advantages of both non-targeted and targeted metabolomics. It can provide a new semi-quantitative fingerprinting with high coverage. We combined this pseudotargeted metabolomic fingerprinting with deep learning technology for the identification and visualization of the pathogen. A variational autoencoder framework was performed to identify and classify pathogenic bacteria and achieve their visualization, with prediction accuracy exceeding 99%. Therefore, this technology will be a powerful tool for rapidly and accurately identifying pathogens.

## Introduction

Most foodborne diseases are related to foodborne pathogens such as *Listeria monocytogenes*, *Salmonella* spp., *Escherichia* spp., *Staphylococcus aureus*, *Enterococcus* spp., *Yersinia enterocolitica*, *Bacillus cereus*, and so forth (Marshall et al., 2020; Sarno et al., 2021). More than 250 foodborne diseases have been identified, and it is estimated that 76 million people are affected by foodborne diseases, causing 5,000 deaths each year in the United States (Dong et al., 2020; Saeed et al., 2021). Methods based on genome sequencing are helpful for this, but their ability to predict traits is limited. Analytical strategies that use the inherent information content of the phenotype to bypass these limitations have been established, for example, the matrix-assisted laser desorption/ionization time-of-flight mass spectrometry (MALDI-TOF-MS) method. However, conventional standard procedures cannot directly distinguish some species with high genetic correlation or with closely related environmental conditions (He et al., 2010; Martiny et al., 2012; Paauw et al., 2015; Ferone et al., 2020), such as *Shigella castellani* and *Escherichia coli* (Khot and Fisher, 2013), *B. pseudomallei*, and *B. thailandensis* (Dingle et al., 2014; Watthanaworawit et al., 2021). It is urgent to develop sensitive and accurate methods to monitor foodborne pathogens stringently.

Metabolomic techniques are screened as a better alternative method that measures compounds with low molecular weight (MW <1,000) directly associated with microbial activity at a given point in time and under specific environmental conditions (Fiehn, 2002). It is proposed that metabolomics technology can become a detection platform for foodborne pathogens and spoilage microorganisms (Pinu, 2016). The pseudotargeted metabolomics method has the advantages of both non-targeted and targeted metabolomics. High-resolution MS is used to obtain the ion pair information of metabolites. Meanwhile, the targeted single-reaction monitoring (SRM) or multiple reaction detection (MRM) method, based on triple quadrupole (QQQ) MS, is used to measure the abundance of metabolites in actual sample analysis. The method not only has high coverage, good linearity, and reproducibility but also does not require standard samples to limit the detected metabolites (Chen et al., 2013; Wang et al., 2016). In addition, QQQ-MS analysis increases stability, further reduces costs, and is conducive to high-throughput sample analysis.

Machine learning (ML) that is the concept of “training” computational methods can improve given more “experience” or data. Convolutional neural network has demonstrated its excellent learning ability in many applications. The design of the convolutional layer for feature extraction is particularly critical in various problems (Ho et al., 2019; Thrift et al., 2020; Sil et al., 2021). Variational autoencoders (VAE) combine neural network and Bayesian theory to learn suitable latent variables from data to represent input data (Thrift et al., 2020). We have established a recognition model based on VAE, which strengthens the representation ability of VAE from network structure. The feature extraction ability of the convolutional neural network is also improved from the perspective of theoretical analysis.

In our work, we combined pseudotargeted metabolomic fingerprinting with deep learning technology to realize the identification of pathogens. We achieved a prediction accuracy of the VAE model that exceeded 99%. This methodology is based on the QQQ-MS detection platform, which has minimal pre-processing steps and high identified accuracy at a lower cost and will be a powerful tool for rapidly and accurately identifying pathogens.

## Materials and Methods

### The Culture Conditions of Bacterial Strains and Sample Preparation

The total strains used in this study are presented in Supplementary Table 1. Brain heart infusion broth was used for bacteria cultivation at 37°C with shaking at 200 rpm until OD_600_ = 0.6 ± 0.05. In each experiment, a single fresh colony was inoculated into a 10-ml medium, cultivated with shaking at 37°C and 200 rpm. After overnight growth, the OD_600_ value was adjusted to about 0.2, and then 50-ul culture was inoculated into 10 ml of medium grown and until its OD_600_ ∼0.6 ± 0.05.

To collect bacterial cells, 1 ml of culture was centrifuged at −10°C and 12,000 rpm for 15 min. After the collected bacteria were washed twice with cold PBS solution, liquid nitrogen was used to quench the metabolism. Then, 800 μl of cold extract solution (acetonitrile/methanol/water = 2:2:1, containing an isotopically labeled internal standard mixture) was added into the samples for ultrasonic disintegration. After ultrasonic decomposition, the samples were incubated at −20°C for 1 h, and the collected supernatant was centrifuged at 12,000 rpm and 4°C for 15 min. Then, the collected supernatant samples were dried in a vacuum. Finally, the dried samples were resuspended in 200 ul acetonitrile solution (acetonitrile/water = 1:1) at room temperature and sonicated for 15 min. The resulting supernatant was transferred to a fresh glass vial for liquid chromatography (LC)/MS analysis. The quality control (QC) sample was prepared by mixing equal aliquots of the supernatants from all of the samples.

### Liquid Chromatography–Mass Spectrometry/Mass Spectrometry Analysis

#### The Untargeted Metabolomics Analysis of Quality Control by Ultra Performance Liquid Chromatography–Quadrupole–Orbitrap–Mass Spectrometry

A Thermo Fisher Scientific UltiMate 3000 Rapid Separation LC system with a ultra performance liquid chromatography (UPLC) HSS T3 column (2.1 mm × 100 mm, 1.8 μm) coupled to a Q Exactive Hybrid Quadrupole-Orbitrap mass spectrometer (Thermo Fisher Scientific) was used to perform the LC–MS/MS analyses. The mobile phase A and the mobile phase B were 0.1% formic acid in water and acetonitrile for positive mode, respectively. The elution gradient was set as follows: 0–1.0 min, 1% B; 1.0–8.0 min, 1–99% B; 8.0–10.0 min, 99% B; 10.0–10.1 min, 99–1% B; 10.1–12 min, 1% B. Its flow rate was 0.5 ml/min, and the injected volume was 2 μl. The acquisition of MS/MS spectra depended on the QE mass spectrometer’s information-dependent acquisition mode. The electron spray ionization (ESI) source conditions were set as follows: sheath gas flow rate as 45 Arb, aux gas flow rate as 15 Arb, capillary temperature of 400°C, full MS resolution as 70,000, MS/MS resolution as 17,500, collision energy as 20/40/60 in NCE mode, and spray voltage as 4.0 kV (positive).

Compound Discoverer (CD) from Thermo Fisher Scientific™ was used for peak detection, extraction, alignment, and integration. Then, in-house and open-source MS2 databases were applied in metabolite annotation. The precursor ion and its intensity as well as the MS2 product ion and its intensity information corresponding to the parent ion under each collision energy were extracted from the raw data by deconvolution. Hundreds of metabolites are available and then removed without secondary MS. After the CD process, a list of precursor, product ion, and each collision energy of metabolites was derived. Then, the list was imported by TSQ method editing template to generate a method with SRM channels used for pseudotargeted metabolomics analysis.

#### Pseudo-Targeted Metabolomics Analysis by Ultra Performance Liquid Chromatography–Electron Spray Ionization–Quadrupole–Mass Spectrometry/Mass Spectrometry

A method with SRM channels was used for pseudotargeted metabolomics analysis. Before analyzing the actual samples, the QC sample was used to calibrate retention times and optimize collision energy and delete ion pairs that MRM analysis cannot gather. Finally, a scheduled SRM method that includes 252 metabolite transitions was constructed using Tracefinder (Thermo Fisher Scientific, United States) and used for actual sample analysis to obtain fingerprints. The chromatographic column, mobile phase, and elution gradient were the same as the above-mentioned UPLC-Q-Orbitrap–MS method. Thermo Fisher Scientific UHPLC coupled with TSQ Quantiva Triple Quadrupole Mass Spectrometer (UPLC-QQQ-MS) equipped with a turbo ion spray source was used with the following mass spectrometer settings in positive ion mode: ion spray voltage = 4,500 V, temperature = 400°C, ion source gas 1 = 30 psi, ion source gas 2 = 70 psi, curtain gas = 20 psi, collision gas = 5 psi, ion spray probe vertical position = 3, and ion spray probe horizontal position = 5.

#### The Architecture of Variational Autoencoder

Deep neural network models were performed in Keras, and its optimizer was Adam (Zhang et al., 2019). The VAE encodes high-dimensional data (LC–MS profiles) into a low-dimensional latent space to select primary representations of the data. It is composed of an encoder network and a decoder network. The encoder network encodes spectra as a Gaussian probability distribution in the n-dimensional latent space, schematically depicted as μ and Σ. The decoder network decodes sample points from the latent space back into the original spectra. The construction of our VAE using deep convolutional neural networks, architecture, and training parameters are as follows:

(1) The first part is used for feature extraction and representation of data, including two convolutional layers. The function of the first-layer convolutional network was feature extraction and data representation, which can be expressed as:


(1) $$F_{1}\,\left(Y\right)=\max\left(0,W_{1}^{\text{⋆}}\,Y+B_{1}\right)$$

where W1 was the convolution kernel, B1 was the deviation of n1 dimension, * represented the convolution operation, W1 expression was *c*1 × f1 × 1, which represented c1 convolution kernel of f1 × 1, and *c*1 was the number of filters (the number of filters in the first layer in this study is set to 256, so *c* = 256). Input sample Y was convolved through a filter (Conv1D) to obtain the eigenvector of Y, and the eigenvector produced by this layer was processed. Output by the ReLU activation functions to obtain F1(Y).

The second layer was expressed as:


(2) $$F_{2}\,\left(Y\right)=\max\left(0,W_{2}^{*}\,F_{1}\,\left(Y\right)+B_{2}\right)$$

(2) The second part is mainly to learn the mean and variance features. F2(Y) is sequentially flattened through the Flatten operation, and the fully connected network was used to learn the features μ and σ, respectively. Then, weighted summation of the two network layers was performed to obtain *z*, which can be expressed as:


(3) $$\mu =W_{3}^{*}\,F\,l\,a\,t\,t\,e\,n\,\left(F_{2}\,\left(Y\right)\right)+B_{3}$$

(4) $$\sigma =W_{4}^{*}\,F\,l\,a\,t\,t\,e\,n\,\left(F_{2}\,\left(Y\right)\right)+B_{4}$$

(5) z=μ+σ⋅ε,ε∼𝒩⁢(0,1)

(3) The third part is data reconstruction, which mainly contains 4 convolutional layers, which were represented, in turn, as:


(6) $$F_{3}\,\left(Y\right)=\max\left(0,W_{5}^{*}\,z+B_{5}\right)$$

(7) $$F_{4}\,\left(Y\right)=\max\left(0,W_{6}^{*}\,F_{3}\,\left(Y\right)+B_{6}\right)$$

(8) $$F_{5}\,\left(Y\right)=\max\left(0,W_{7}^{*}\,F_{4}\,\left(Y\right)+B_{7}\right)$$

(9) $$F_{6}\,\left(Y\right)=\max\left(0,W_{8}^{*}\,F_{5}\,\left(Y\right)+B_{8}\right)$$

where the convolution kernel was W1, the deviation was Bi, the number of filters was 1, 32, 64, and 1, respectively, and the activation functions were all Relu functions. The loss function of the VAE network was usually defined as the sum loss of L2 and KL divergence. The L2 loss is mainly responsible for calculating the reconstruction error, and the KL divergence loss is mainly responsible for the distribution error. Since the distribution of the sample curve had a low impact on the classification results, we had used the L2 loss alone as the loss function of the network. After the training of the VAE network was completed, a fully connected layer with 256 neurons is added separately to the back end of the z layer, which can be expressed as follows:


(10) $$F_{7}\,\left(Y\right)=W_{9}^{*}\,F_{6}\,\left(Y\right)+B_{9}$$

Since this study deals with 6 classification problems, the number of neurons in the second fully connected layer was set to 6. F7(Y) and get F8(Y) were entered after processing the Softmax activation function. F8(Y) was the classification result of the sample.

(4) Accuracy was used as the loss function of the network algorithm in this study to learn the parameters of the network. The essence of the training was to optimize these parameters. Accuracy can be defined as follows:


(11) $$\text{Accuracy}=\frac{T\,n}{N}$$

In this formula, *N* denoted the number of training samples, and Tn denoted the number of training samples with correct prediction.

The size of the convolution kernel, the number of convolution kernels, the step size during convolution, and the size of zero-fill were all parameters set for each layer. Supplementary Table 2 displays the detailed data records.

## Results and Discussion

### Pseudotargeted Profiles Obtained by Liquid Chromatography–Mass Spectrometry Analysis

**FIGURE 1:**
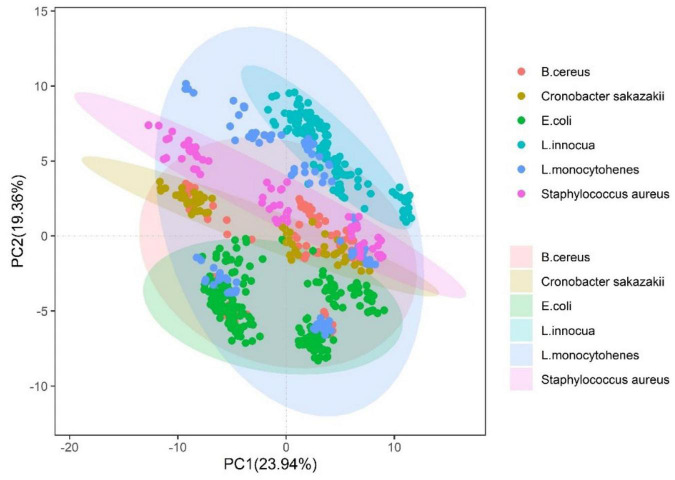
Principal component analysis of common pathogens from *Bacillus cereus*, *Escherichia coli*, *Enterobacter sakazakii*, *Listeria innocua*, *Listeria monocytohenes*, and *Staphylococcus aureus.*

**FIGURE 2:**
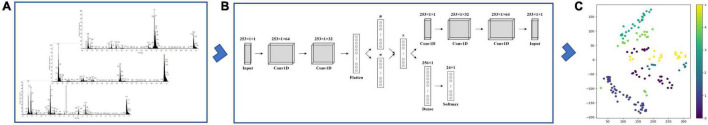
A variational autoencoder (VAE) for common foodborne pathogen classification. **(A)** The selected pseudo-targeted LC–MS profiles. We selected 253 ion pairs from the untargeted strategy and detected them in actual pathogen samples using the UHPLC/QQQ MRM MS-based pseudotargeted metabolomics method. **(B)** The schematic of the VAE model. The VAE is composed of an encoder network and a decoder network. The encoder network encodes spectra into a Gaussian probability distribution in the n-dimensional latent space, and the decoder network decodes sample points from the latent space back into the original spectra. The encoder and decoder networks used deep convolutional neural network. **(C)** Visualization of prediction results.

The pseudotargeted metabolomic was analyzed using a UHPLC/QQQ–MS system operated in the MRM mode. The MRM ion pairs were selected from the Q Exactive Hybrid Quadrupole-Orbitrap mass spectrometer system through untargeted tandem MS of the real QC samples. As a proof of concept, 253 metabolites were qualified, as can be seen in Supplementary Table 3. Finally, these ion pairs were detected using the UHPLC/QQQ MRM MS-based pseudotargeted metabolomics method, as shown in Figure 2A. We acquired the LC–MS pseudo-targeted profile of the samples within 12 min as a training dataset, as shown in Figure 2B. It is ensured that each individual spectrum was taken from a single strain culture, and the preparation conditions were consistent between samples (detailed in section “Materials and Methods”). We constructed reference datasets of over 828 spectra from 22 bacterial strains for two batches. Each batch and parallel samples were independent of each other in over 10 parallel samples. The total samples were divided into two parts: the training set and the validation set. One was used to the model trained, and another was used to confirm the recognition ability of the method and the prediction of unknown strains. The distribution of strains in the training set used principal component analysis, as shown in Figure 1. It can be seen that the strains cannot be separated when used in its dimensionality reduction analysis.

### Deep Neural Network Models for Common Foodborne Pathogen Classification

The equations should be inserted in editable format from the equation editor. The dataset including the pseudotargeted metabolomic fingerprints of cell lysate from *B. cereus*, *E. coli*, *E. sakazakii*, *L. innocua*, *L. monocytogenes*, and *S. aureus* was used to train a VAE framework, as can be seen in Supplementary Table 4. Variational autoencoder architecture (VAE) was implemented to derive compact data representations and analyze valuable predictors, respectively. Figure 2 depicts the scheme using a VAE for common foodborne pathogen classification. The VAE works by capturing the necessary representation of data by encoding high-dimensional data points into a low-dimensional latent space. The VAE is composed of an encoder network and a decoder network. The encoder network encodes spectra into a Gaussian probability distribution in the n-dimensional latent space, and the decoder network decodes sample points from the latent space back into the original spectra. Our constructed VAE using deep convolutional neural networks, architecture, and training parameters is further described in section “Materials and Methods.” There are three advantageous features in our VAE that have been provided by encoding spectra as probability distributions in a lower-dimensional latent space: (1) improve the prediction capabilities of classification. Due to a well-structured latent space in VAE, simple models can also make predictions from the encoded data. VAE encodes each spectrum as a distribution, and these distributions could overlap. If the overlapping distributions were not from a similar spectrum, the model was severely penalized during the training process; (2) predictions made from models trained on encoded data were improved by de-noising, especially for small amounts of labeled data; and (3) VAE was able to visualize variations in the latent space and decode the spectrum due to the continuously represented coded distributions of latent space.

**FIGURE 3:**
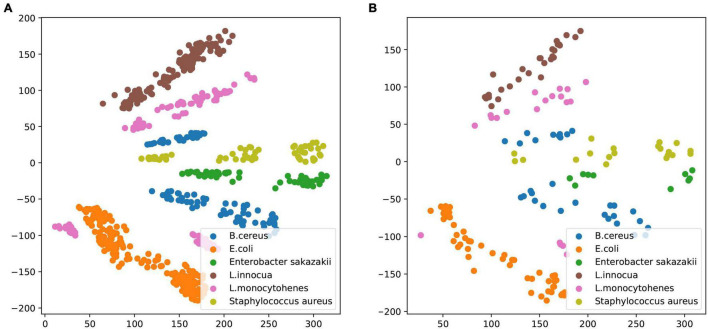
Variational autoencoder space of common pathogens from *Bacillus cereus*, *Escherichia coli*, *Enterobacter sakazakii*, *Listeria innocua*, *Listeria monocytohenes*, and *Staphylococcus aureus*. **(A)** The trained dataset was used to train the model. **(B)** The validation dataset was used to test the model.

Prior to training the VAE, bacterial cultures were prepared, and their LC–MS profiles were collected as described in section “Materials and Methods.” It is ensured that each individual spectrum was taken from a single strain culture, and the preparation conditions were consistent between samples. Then, a training dataset of about 669 samples and a validation dataset of about 160 samples were used to realize the classification and identification of common pathogens by VAE. The model was trained on a training dataset and tested on an independent validation dataset gathered from separately cultured samples. Common pathogens included *B. cereus*, *E. coli*, *C. sakazakii*, *L. innocua*, *L. monocytohenes*, and *S. aureus*. The encoded pseudotargeted metabolomic profiles from cellular lysate are plotted in Figure 3. The LC–MS profile spectra were quantitatively analyzed to obtain trained and validation datasets, respectively. After normalization and standardization, the trained dataset was encoded using VAE, which trained with the spectral dataset from bacterial lysate under experimental conditions. Although the Figure 3 plots were arbitrarily rotated around VAE axes, the differences in the relative location of data in the VAE latent space represent corresponding changes in the characteristics of features. Thus, it is notable that the difference in VAE values concerning different species of common pathogens demonstrates that this approach can successfully distinguish common pathogens at the species level. The pathogen LC–MS profiles used the VAE method to analyze the results of the trained dataset as shown in Figure 3A, and the results of the validated dataset are shown in Figure 3B.

**FIGURE 4:**
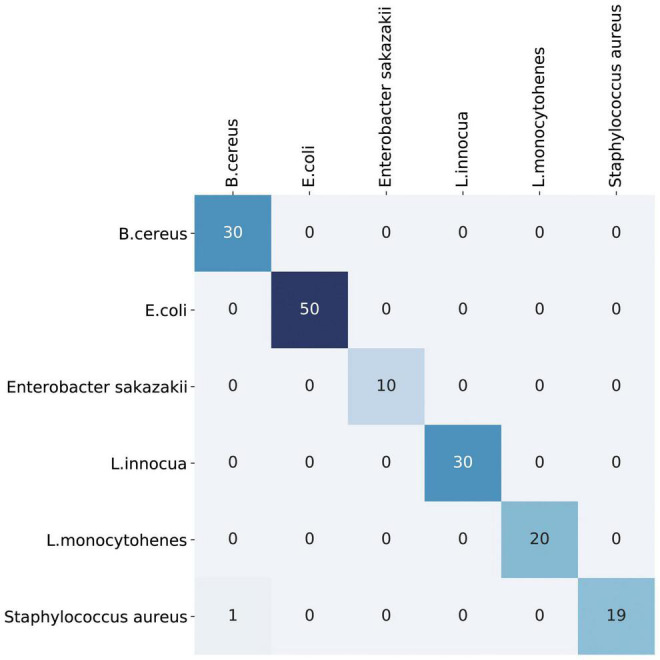
The confusion matrix chart of the validated dataset predicted the results. Its predicted accuracy of identification exceeded 99%.

We also evaluated the use of support vector machines and simple CNN architectures, but the VAE showed the best performance. Compared with the support vector machine and simple CNN models, the VAE model significantly improved the classification and prediction ability of the training set and could visualize variations in the entire latent space as well. The prediction accuracy of the validation dataset exceeded 99%, as shown in Figure 4 and Table 1. The prediction accuracy rates of the support vector machine and CNN models for the validation dataset were 93.13 and 98.75%, respectively (see Supplementary Figure 1).

**TABLE 1:** Prediction accuracy of each type of pathogens.

Pathogens	Predicted accuracy
*Bacillus cereus*	100%
*Escherichia coli*	100%
*Enterobacter sakazakii*	100%
*Listeria innocua*	100%
*Listeria monocytohenes*	100%
*Staphylococcus aureus*	95%
Total	99.38%

## Discussion

In LC–MS/MS-based investigation, metabolite fingerprints are described by retention time, *m*/*z* values, and corresponding intensities of detected ions. In normal fingerprinting, the chemical structure of the detected metabolites typically remains unknown, and the quantitative information is not contained. Pseudotargeted metabolomics realizes quantitative analysis with both high coverage and high performance of quantitative analysis (Zheng et al., 2020). In this study, a new deep semi-quantitative metabolic fingerprinting that was obtained using the pseudotargeted metabolomics method was applied to identify and classify common pathogens. We used the VAE–CNN model that combined pseudotargeted metabolomics technology and deep learning technology to realize the identification of foodborne pathogens and the visualization of classification. As illustrated in Figure 3, the deep learning model successfully differentiated the common foodborne samples. However, to verify this method under a broader range of samples and conditions, we plan to collect more strains at different concentrations and optimize the model to shorten the fingerprint time further. In addition, we will study the effectiveness of metabolomics technology in identifying and distinguishing pathogenic and non-pathogenic foodborne pathogens in food. Furthermore, whether the emergence of multiple microbial species under these conditions affects their fingerprint expression will also be investigated. This technology, based on the QQQ-MS detection platform, has the merit of higher typing and identification accuracy at a lower cost, which is expected to replace the MALDI-TOF method based on TOF high-resolution MS. The application of this methodology may significantly reduce the analysis time required to detect and confirm these important foodborne pathogens.

## Conclusion

We combined pseudotargeted metabolomic fingerprinting with the VAE framework to successfully realize the identification and visualization of pathogens. The prediction accuracy of the VAE model that we achieved was over 99%. This technology is based on the QQQ-MS detection platform, which has minimal pre-processing steps, showing high accuracy at a lower cost. It is a powerful tool that will be used to replace the MALDI-TOF method for the rapid and accurate identification of pathogens.

## Data Availability Statement

The original contributions presented in the study are included in the article/Supplementary Material, further inquiries can be directed to the corresponding authors.

## Author Contributions

QW, YD, and YF conceived and designed the study, and reviewed the manuscript. YF performed the experiments, wrote the manuscript, and analyzed the results. MoC, XW, HZ, YZ, JZ, LX, MiC, LH, and GC isolated and collected strains. All authors contributed to the article and approved the submitted version.

## Conflict of Interest

The authors declare that the research was conducted in the absence of any commercial or financial relationships that could be construed as a potential conflict of interest.

## Publisher’s Note

All claims expressed in this article are solely those of the authors and do not necessarily represent those of their affiliated organizations, or those of the publisher, the editors and the reviewers. Any product that may be evaluated in this article, or claim that may be made by its manufacturer, is not guaranteed or endorsed by the publisher.
