# Supplementary material for: Pseudotargeted Metabolomic Fingerprinting and Deep Learning for Identification and Visualization of Common Pathogens
Source: Front Microbiol. 2022 Mar 10;13:830832. doi: 10.3389/fmicb.2022.830832 (PMC8960985; doi:10.3389/fmicb.2022.830832)
Supplement: Supplementary file 1 [file Data_Sheet_1.docx]

Supplementary Material

# Supplementary Table 1. Pathogen strains information of this study.

| Bacterial species | Strains | Number of strains | Source |
| --- | --- | --- | --- |
| *L. monocytogenes*  *L.innocua*  *Escherichia coli*  *Staphylococcus aureus*  *Cronobacter sakazakii*  *B.cereus*  Total | 1834-2  1962-1  2103-1  85-1  ^1^NCTC 11288  1969  2120  2016  2941C  ^2^ATCC 44102  ATCC 8099  ^3^CMCC44103  ATCC8739  25922  ATCC 29213  21593  1541AH  2991W  1408  14579  63301 | 4  5  5  2  2  3  21 | a  a  a  a  a  a  a  a  a  a  a  a  a  a  b  a  a  a  a  a  a |

a, our laboratory;

b, Guangdong Huankai Co., Ltd., China;

^1^NCTC, National Collection of Type Cultures, UK.

^2^ATCC, American Type Culture Collection, USA.

^3^CMCC, National Center for Medical Culture Collections, China

# Supplementary Table 2. Parameter settings for each layer

| Name | Layer parameter | | | |
| --- | --- | --- | --- | --- |
|  | Channel | Kernel size | Stride | Padding |
| Con1D_1 | 64 | 5×1 | 1 | 0 |
| Con1D_2 | 32 | 5×1 | 1 | 0 |
| Flatten | 1 | 1×1 | 1 | 0 |
| Dense_μ | 2-d fc, Relu | | | |
| Dense_σ | 2-d fc, Relu | | | |
| Layer_ z | 2-d fc, Relu | | | |
| Dense2Con1D | 253×1×1, Relu | | | |
| Con1D_3 | 32 | 5×1 | 1 | 0 |
| Con1D_4 | 64 | 5×1 | 1 | 0 |
| Con1D_5 | 1 | 5×1 | 1 | 0 |

| **Supplementary Table 3. 253 qualitative metabolites.** | | | | | | | | | |
| --- | --- | --- | --- | --- | --- | --- | --- | --- | --- |
|  | Metabolite Name |  | Molecular Weight | ExtractedMass | Adduct | RT | Fragment | Fragment | Fragment |
| 1 | MW_110.1096 | C8 H14 | 110.1096 | 111.1168 | M+H | 8.64 | 70.0652 | 69.0700 | 112.0507 |
| 2 | MW_156.1263 | C8H16N2O | 156.1263 | 189.1597 | M+H | 0.60 | 72.0808 | 100.0756 | 171.1490 |
| 3 | MW_138.0793 | C7H10N2O | 138.0793 | 139.0866 | M+H | 3.36 | 56.9650 | 69.0448 | 140.0683 |
| 4 | MW_128.0951 | C6H12N2O | 128.0951 | 161.1286 | M+H+MeOH | 0.60 | 103.0390 | 60.0810 | 85.0284 |
| 5 | ({5-[(2S)-2-Carboxy-2-(trimethylammonio)ethyl]-1H-imidazol-2-yl}sulfanyl)oxidanide | C9H15N3O3S | 245.0834 | 246.0908 | M+H | 0.80 | 70.0652 | 71.0685 | 148.0602 |
| 6 | (2S)-5-Carbamimidamido-2-(2-oxo-1-azetidinyl)pentanoic acid | C9H16N4O3 | 228.1222 | 229.1296 | M+H | 0.76 | 70.0652 | 229.1294 | 116.0706 |
| 7 | (Ac)2-L-Lys-D-Ala | C13H23N3O5 | 301.1637 | 302.1711 | M+H | 2.99 | 86.0600 | 189.0870 | 86.0964 |
| 8 | α-Aminoadipic acid | C6H11NO4 | 161.0688 | 162.0760 | M+H | 0.89 | 103.0390 | 60.0810 | 85.0284 |
| 9 | Coniceine | C8 H15 N | 125.1205 | 126.1278 | M+H | 3.89 | 84.9598 | 102.9703 | 56.9651 |
| 10 | δ-Valerolactam | C5H9NO | 99.0684 | 100.0757 | M+H | 3.10 | 56.9650 | 56.0497 | 76.5151 |
| 11 | PEG n10 | C19H34N4O6 | 414.2462 | 432.2799 | M+NH4 | 3.98 | 89.0597 | 133.0859 | 415.2531 |
| 12 | linatine | C10H17N3O5 | 259.1167 | 260.1241 | M+NH4 | 0.83 | 86.0600 | 189.0869 | 260.1237 |
| 13 | 1-(β-D-ribofuranosyl)thymine | C10H14N2O6 | 258.0852 | 259.0925 | M+H | 0.82 | 86.0601 | 189.0870 | 260.1242 |
| 14 | 1,3-Benzodioxolylbutanamine | C11H15NO2 | 193.1103 | 194.1174 | M+H | 3.59 | 135.0439 | 58.0653 | 107.0491 |
| 15 | 1,3-diazaspiro[4.5]decane-2,4-dione | C8H12N2O2 | 168.0899 | 169.0972 | M+H | 3.15 | 70.0652 | 169.0971 | 170.0968 |
| 16 | 1-[(1-methyl-1H-imidazol-4-yl)sulfonyl]-4-(2-pyridinyl)piperazine | C13H17N5O2S | 285.1324 | 308.1216 | M+Na | 3.17 | 237.0843 | 309.1280 | 140.0317 |
| 17 | 1-desoxymethylsphinganine | C17H37NO | 301.2979 | 302.3052 | M+H | 6.37 | 88.0757 | 70.0652 | 106.0862 |
| 18 | 1-palmitoyl-2-hydroxy-sn-glycero-3-phosphoethanolamine | C21H44NO7P | 453.2852 | 454.2924 | M+H | 7.47 | 313.2735 | 57.0701 | 62.0602 |
| 19 | 1-Pentofuranosyl-2,4(1H,3H)-pyrimidinedione | C9H12N2O6 | 244.0695 | 262.1032 | M+NH4 | 0.70 | 146.1175 | 164.9299 | 87.0440 |
| 20 | 1-Piperideine | C5H9N | 83.0735 | 84.0808 | M+H | 0.53 | 68.1295 | 85.1322 | 66.1155 |
| 21 | 2-(Cyclohexylmethylidene)-1,2,3,4-tetrahydronaphthalen-1-one | C17H20O | 240.1474 | 241.1546 | M+H | 2.77 | 58.0654 | 242.1577 | 84.0808 |
| 22 | 2,3,4,9-Tetrahydro-1H-β-carboline-3-carboxylic acid | C12H12N2O2 | 216.0899 | 217.0972 | M+H | 3.85 | 144.0807 | 145.0842 | 217.0969 |
| 23 | 2,4,5-Trimethylaniline | C9H13N | 135.1048 | 136.1121 | M+H | 11.29 | 91.0542 | 137.1158 | 121.0886 |
| 24 | 2,4,6-triaminotoluene | C7H11N3 | 137.0953 | 138.1026 | M+H | 11.27 | 56.0498 | 98.0476 | 98.0713 |
| 25 | 2,4-DIMETHYL-2-IMIDAZOLINE | C5H10N2 | 98.0844 | 99.0916 | M+H | 10.90 | 81.0447 | 99.0554 | 57.0449 |
| 26 | 2,6-Diamino-4-hexenoic acid | C6H12N2O2 | 144.0899 | 145.0971 | M+H | 0.91 | 87.0440 | 146.1174 | 60.0809 |
| 27 | 2-[(2-chlorobenzyl)sulfanyl]-4,6-dimethylnicotinonitrile | C15H13ClN2S | 326.0006 | 327.0079 | M+H | 6.69 | 98.9842 | 174.9921 | 116.9947 |
| 28 | 2-Amino-1,3,4-octadecanetriol | C18H39NO3 | 317.2928 | 318.3001 | M+H | 5.89 | 256.2632 | 88.0757 | 319.3034 |
| 29 | 2-Amino-4-methylpyrimidine | C5H7N3 | 109.0640 | 110.0710 | M+H | 0.57 | 83.0603 | 93.0447 | 87.0040 |
| 30 | 2-amino-N-(1,3,5-trimethyl-1H-pyrazol-4-yl)benzamide | C13H16N4O | 222.1468 | 245.1360 | M+Na | 3.85 | 86.0964 | 70.0652 | 246.2063 |
| 31 | 2-Mercaptoethanol | C2H6OS | 78.0139 | 79.0212 | M+H | 0.89 | 63.9978 | 56.9650 | 61.0108 |
| 32 | 2-Oxobutyric acid | C4H6O3 | 102.0317 | 103.0390 | M+H | 0.66 | 60.0810 | 84.9597 | 105.1103 |
| 33 | 3-(2-methylpropyl)-octahydropyrrolo[1,2-a]pyrazine-1,4-dione | C11H18N2O2 | 210.1368 | 211.1440 | M+H | 4.36 | 70.0652 | 95.0492 | 141.0004 |
| 34 | 3-(3,4-dihydroxyphenyl)propanoic acid | C9H10O4 | 164.0474 | 165.0547 | M+H | 1.12 | 123.0440 | 95.0491 | 119.0491 |
| 35 | 3-(propan-2-yl)-octahydropyrrolo[1,2-a]pyrazine-1,4-dione | C10H16N2O2 | 196.1212 | 197.1286 | M+H | 3.85 | 70.0652 | 131.9742 | 72.9372 |
| 36 | 3,6,9,12-tetraoxatridecan-1-ol | C9H20O5 | 208.1311 | 231.1203 | M+Na | 3.53 | 70.0651 | 72.0809 | 86.0964 |
| 37 | 3-[hydroxy(oxido)phosphoranyl]pyruvic acid | C3H5O5P | 151.9876 | 152.9949 | M+H | 11.37 | 70.9418 | 97.0095 | 129.9789 |
| 38 | 3-amino-2,3-dideoxy-scyllo-inosose | C6H11NO4 | 161.0688 | 162.0761 | M+H | 0.80 | 84.0444 | 103.0389 | 60.0809 |
| 39 | 3-amino-2-phenyl-2H-pyrazolo[4,3-c]pyridine-4,6-diol | C12H10N4O2 | 242.0804 | 243.0877 | M+H | 4.77 | 172.0869 | 216.0767 | 244.0909 |
| 40 | 3-ethyl 5-methyl 2-[(1,3-benzodioxol-5-ylmethyl)amino]-6-methylpyridine-3,5-dicarboxylate | C19H20N2O6 | 372.1384 | 373.1457 | M+H | 3.06 | 70.0652 | 216.0717 | 374.1483 |
| 41 | 3-Hydroxybenzaldehyde | C7H6O2 | 122.0368 | 123.0441 | M+H | 2.05 | 80.0495 | 95.0492 | 96.0444 |
| 42 | 3-Hydroxy-DL-kynurenine | C10H12N2O4 | 242.0905 | 225.0870 | M+H-H2O | 2.82 | 125.0710 | 70.0652 | 84.0808 |
| 43 | 3-Methylcyclopentene | C6H10 | 82.0782 | 83.0855 | M+H | 11.29 | 56.0497 | 55.0545 | 84.0808 |
| 44 | 4-Amino-2-(4-pyridyl)pyrimidine-5-carbothioamide | C10H9N5S | 209.0722 | 232.0616 | M+Na | 0.74 | 144.9930 | 233.0644 | 250.0050 |
| 45 | 4-Hydroxyprolylleucine | C11H20N2O4 | 244.1423 | 245.1497 | M+H | 2.99 | 132.0655 | 86.0964 | 86.0601 |
| 46 | 4-Methyleneglutamic acid | C6H9NO4 | 159.0532 | 160.0605 | M+H | 0.89 | 101.0597 | 60.0810 | 55.0545 |
| 47 | 4-Piperidinecarboxamide | C6H12N2O | 96.0687 | 129.1022 | M+H+MeOH | 0.91 | 84.0444 | 130.0499 | 84.0808 |
| 48 | 5,6-Dihydrothymidine | C10H16N2O5 | 244.1059 | 245.1132 | M+H | 0.89 | 70.0652 | 127.0865 | 155.0814 |
| 49 | 5-Nitro-2-propoxyaniline | C9H12N2O3 | 196.0849 | 229.1184 | M+H+MeOH | 0.89 | 100.0757 | 230.1862 | 114.0914 |
| 50 | 5'-S-Methyl-5'-thioadenosine | C11H15N5O3S | 297.0895 | 298.0968 | M+H | 3.45 | 136.0617 | 137.0661 | 61.0108 |
| 51 | 6-(3-chloro-4-methylphenyl)-1,3,5-triazine-2,4-diamine | C10H10ClN5 | 235.0641 | 236.0714 | M+H | 0.56 | 201.3207 | 153.8827 | 165.8476 |
| 52 | 6-(4-phenylpiperazino)hexanoic acid hydrochloride | C16H24N2O2 | 254.1993 | 277.1886 | M+Na | 5.86 | 250.0053 | 278.1913 | 231.9949 |
| 53 | 8-(1,2-dihydroxy-3-methylbut-3-en-1-yl)-7-methoxy-2H-chromen-2-one | C15H16O5 | 276.0957 | 277.1030 | M+H | 0.82 | 86.0600 | 189.0870 | 276.1191 |
| 54 | 9(Z),11(E),13(E)-Octadecatrienoic Acid methyl ester | C19H32O2 | 298.2507 | 299.2579 | M+H | 8.49 | 57.0701 | 71.0856 | 95.0855 |
| 55 | Aceglutamide | C7H12N2O4 | 188.0796 | 189.0870 | M+H | 0.77 | 129.1020 | 130.0860 | 86.0600 |
| 56 | Acetylcholine | C7H15NO2 | 145.1102 | 146.1175 | M+H | 0.72 | 87.0440 | 146.1175 | 60.0810 |
| 57 | ACPC | C4H7NO2 | 101.0477 | 84.0444 | M+H-H2O | 0.64 | 68.1295 | 85.1322 | 66.1155 |
| 58 | Adenine | C5H5N5 | 135.0545 | 136.0617 | M+H | 0.92 | 119.0352 | 137.0661 | 94.0400 |
| 59 | Adenine | C5H5N5 | 135.0545 | 136.0618 | M+H | 3.45 | 91.0542 | 95.0492 | 137.0460 |
| 60 | Adenosine | C10H13N5O4 | 267.0965 | 268.1039 | M+H | 1.67 | 136.0620 | 119.0351 | 268.1030 |
| 61 | Adenosine 5'-monophosphate | C10H14N5O7P | 347.0626 | 348.0699 |  | 0.94 | 136.0620 | 97.0283 | 69.0335 |
| 62 | AL 8810 | C24H31FO4 | 424.2069 | 425.2140 | M+H | 8.53 | 365.1927 | 89.0597 | 239.1489 |
| 63 | Allopurinol | C5H4N4O | 136.0385 | 137.0458 | M+H | 1.56 | 91.0542 | 119.0352 | 137.0641 |
| 64 | alpha-Glutamyl-4-hydroxyproline | C10H16N2O6 | 260.1009 | 261.1080 | M+H | 0.84 | 86.0600 | 189.0870 | 70.0652 |
| 65 | Aminocaproic acid | C6H13NO2 | 131.0946 | 132.1019 | M+H | 0.75 | 86.0600 | 68.0495 | 132.0653 |
| 66 | Amobarbital | C11H18N2O3 | 226.1317 | 227.1390 | M+H | 3.91 | 86.0964 | 86.0600 | 68.0496 |
| 67 | Aniline | C6H7N | 93.0578 | 94.0652 | M+H | 0.91 | 53.0389 | 105.0447 | 96.0525 |
| 68 | Aprobarbital | C10H14N2O3 | 210.1005 | 211.1077 | M+H | 3.05 | 70.0650 | 95.0490 | 114.0551 |
| 69 | asn-pro | C9H15N3O4 | 229.1064 | 230.1136 | M+H | 2.90 | 70.0652 | 142.0863 | 184.1081 |
| 70 | asp-leu | C10H18N2O5 | 246.1213 | 247.1289 | M+H | 3.22 | 74.0240 | 86.0960 | 201.1230 |
| 71 | Betaine | C5H11NO2 | 117.0789 | 118.0862 | M+H | 0.63 | 58.0650 | 59.0730 |  |
| 72 | Betaine | C5H11NO2 | 117.0789 | 118.0861 | M+H | 0.72 | 72.0810 |  |  |
| 73 | Biacetyl | C4H6O2 | 86.0368 | 87.0441 | M+H | 0.72 | 68.1295 | 85.1322 | 66.1155 |
| 74 | Bis(4-ethylbenzylidene)sorbitol | C24H30O6 | 414.2038 | 415.2110 | M+H | 6.87 | 119.0856 | 120.0889 | 91.0543 |
| 75 | Bisucaberin | C18H32N4O6 | 400.2319 | 401.2392 | M+H | 3.71 | 86.0600 | 288.1552 | 401.2390 |
| 76 | Butabarbital | C10H16N2O3 | 212.1160 | 245.1495 | M+H+MeOH | 0.91 | 70.0652 | 127.0865 | 155.0814 |
| 77 | Butylamine | C4H11N | 73.0892 | 74.0964 | M+H | 8.94 | 56.0497 | 58.0289 | 57.0337 |
| 78 | Caprolactam | C6H11NO | 113.0841 | 131.1179 |  | 3.71 | 84.0440 | 84.0810 | 72.9371 |
| 79 | Choline | C5H13NO | 103.0997 | 104.1070 | M+H | 0.63 | 60.0810 | 105.1102 | 58.0653 |
| 80 | L-(+)-Citrulline | C6H13N3O3 | 175.0957 | 176.1030 | M+H | 0.65 | 70.0652 | 60.0558 | 116.0706 |
| 81 | cis-4-Hydroxy-D-proline | C5H9NO3 | 131.0582 | 132.0656 | M+H | 3.18 | 68.0500 | 86.0600 |  |
| 82 | cloxyquin | C9H6ClNO | 179.0146 | 180.0218 | M+H | 11.35 | 56.0134 | 97.0397 | 179.0927 |
| 83 | Coniine | C8H17N | 127.1361 | 128.1434 | M+H | 11.29 | 83.0855 | 55.0545 | 129.1468 |
| 84 | Coprine | C8H14N2O4 | 202.0954 | 203.1026 | M+H | 0.77 | 118.0863 | 119.0898 | 85.1323 |
| 85 | Creatinine | C4H7N3O | 113.0590 | 114.0662 | M+H | 11.28 | 72.9372 | 71.0292 | 80.0343 |
| 86 | Cyclo(phenylalanyl-prolyl) | C14H16N2O2 | 244.1211 | 245.1285 | M+H | 4.53 | 120.0807 | 70.0652 | 245.1282 |
| 87 | Cyclohexylamine | C6H13N | 99.1048 | 100.1121 | M+H | 11.30 | 56.9650 | 100.1121 | 103.4881 |
| 88 | Cyprodenate | C13H25NO2 | 227.1885 | 228.1959 | M+H | 6.12 | 95.0855 | 81.0699 | 67.0544 |
| 89 | Cytidine | C9H13N3O5 | 243.0855 | 244.0928 | M+H | 0.91 | 70.0652 | 127.0866 | 155.0814 |
| 90 | Cytosine | C4H5N3O | 111.0432 | 112.0505 | M+H | 0.72 | 95.0240 | 84.0806 | 69.0447 |
| 91 | D-(+)-Proline | C5H9NO2 | 115.0633 | 116.0706 | M+H | 0.72 | 70.0651 | 116.0705 | 71.0685 |
| 92 | D-(+)-Pyroglutamic Acid | C5 H7NO3 | 129.0426 | 130.0498 | M+H | 0.90 | 84.0443 | 130.0498 | 84.0807 |
| 93 | D-Alanine methyl ester | C4H9NO2 | 103.0633 | 104.0706 | M+H | 0.82 | 60.0810 | 84.9596 | 105.1102 |
| 94 | D-Alanyl-D-alanine | C6H12N2O3 | 160.0847 | 161.0920 | M+H | 0.65 | 90.0550 | 96.0524 | 106.0482 |
| 95 | Daminozide | C6H12N2O3 | 160.0848 | 161.0921 | M+H | 0.71 | 103.0390 | 60.0810 | 85.0284 |
| 96 | dapdiamide A | C12H20N4O5 | 300.1431 | 301.1504 | M+H | 0.99 | 70.0652 | 127.0865 | 155.0813 |
| 97 | Desaminotyrosine | C9H10O3 | 166.0631 | 167.0703 | M+H | 7.15 | 84.9597 | 102.9702 | 168.1244 |
| 98 | Diaminopimelic acid | C7H14N2O4 | 190.0953 | 191.1026 | M+H | 0.67 | 82.0650 | 128.0705 | 84.0808 |
| 99 | Diaveridine | C13H16N4O2 | 238.1416 | 261.1310 | M+Na | 3.44 | 70.0652 | 86.0602 | 172.9524 |
| 100 | Diethylamine | C4H11N | 73.0892 | 74.0964 | M+H | 11.25 | 56.0496 | 56.3096 |  |
| 101 | Dihydrothymine | C5H8N2O2 | 128.0586 | 129.0659 | M+H | 0.91 | 84.0444 | 130.0499 | 84.0808 |
| 102 | Dinitrosopentamethylenetetramine | C5H10N6O2 | 186.0869 | 187.0942 | M+H | 3.26 | 86.0601 | 57.0701 | 70.0653 |
| 103 | dinotefuran | C7H14N4O3 | 202.1065 | 203.1139 | M+H | 0.88 | 70.0652 | 144.0656 | 118.0863 |
| 104 | DL-Alanine | C3H7NO2 | 89.0477 | 90.0550 | M+H | 0.54 | 72.0810 | 90.0550 | 90.0914 |
| 105 | DL-Arginine | C6H14N4O2 | 174.1116 | 175.1188 | M+H | 0.52 | 116.0710 |  |  |
| 106 | DL-Glutamine | C5H10N2O3 | 146.0691 | 147.0764 | M+H | 0.62 | 84.0444 | 102.0549 | 130.0499 |
| 107 | DL-Homoserine | C4H9NO3 | 87.0320 | 120.0655 |  | 0.54 | 74.0600 | 56.0497 | 102.0548 |
| 108 | DL-Pipecolinic acid | C6H11NO2 | 129.0790 | 130.0863 | M+H | 0.91 | 84.0444 | 130.0498 | 84.0807 |
| 109 | DL-Serine | C3H7NO3 | 105.0426 | 106.0499 | M+H | 0.53 | 88.0390 | 60.0445 | 106.1115 |
| 110 | DL-Stachydrine | C7H13NO2 | 143.0945 | 144.1018 | M+H | 0.61 | 58.0650 | 59.0731 | 96.0524 |
| 111 | DL-Stachydrine | C7H13NO2 | 143.0945 | 144.1018 | M+H | 0.73 | 60.0810 | 84.9600 | 85.0280 |
| 112 | DL-Tryptophan | C11H12N2O2 | 204.0899 | 205.0971 | M+H | 3.53 | 188.0705 | 146.0599 | 118.0651 |
| 113 | D-Panthenol | C9H19NO4 | 205.1314 | 228.1207 | M+Na | 3.28 | 70.0650 | 141.0000 |  |
| 114 | Drotaverine | C24H31NO4 | 397.2250 | 398.2324 | M+H | 8.87 | 149.0233 | 240.1016 | 399.2359 |
| 115 | Dulcin | C9H12N2O2 | 180.0899 | 213.1234 | M+H+MeOH | 0.93 | 141.0004 | 95.0492 | 158.0270 |
| 116 | Edoxudine | C11H16N2O5 | 256.1058 | 257.1131 | M+H | 3.78 | 70.0652 | 257.1127 | 114.0550 |
| 117 | Eglumetad | C8H11NO4 | 185.0689 | 203.1026 | M+NH4 | 0.91 | 70.0652 | 144.0655 | 118.0862 |
| 118 | epsilon-(gamma-Glutamyl)-lysine | C11H21N3O5 | 275.1481 | 276.1553 | M+H | 0.89 | 147.0763 | 160.1332 | 84.0444 |
| 119 | Erucamide | C22H43NO | 320.3076 | 338.3413 | M+NH4 | 10.81 | 69.0700 | 83.0855 | 57.0701 |
| 120 | Ethephon | C2H6ClO3P | 143.9749 | 144.9821 | M+H | 11.29 | 121.9661 | 88.0231 | 62.9292 |
| 121 | ethyl 2-(5-hydroxy-3-methyl-1-phenyl-1H-pyrazol-4-yl)acetate | C14H16N2O3 | 260.1160 | 261.1232 | M+H | 4.00 | 120.0808 | 261.1233 | 86.0601 |
| 122 | FG7175000 | C9H7NO | 145.0528 | 146.0601 | M+H | 3.56 | 122.0192 | 84.9597 | 97.0076 |
| 123 | Fomepizole | C4H6N2 | 82.0531 | 83.0604 | M+H | 0.70 | 68.1295 | 85.1322 | 84.0444 |
| 124 | Gabapentin | C9H17NO2 | 171.1258 | 172.1331 | M+H | 4.46 | 72.0444 | 89.0710 | 148.9770 |
| 125 | galegine | C6H13N3 | 127.1109 | 128.1182 | M+H | 11.24 | 83.0855 | 55.0545 | 129.1468 |
| 126 | gamma-Glu-gln | C10 H17N3O6 | 275.1117 | 276.1190 | M+H | 0.79 | 86.0601 | 189.0870 | 276.1191 |
| 127 | gamma-Glutamylglutamine | C9H17NO2 | 171.1258 | 172.1331 | M+H | 4.46 | 72.0444 | 89.0710 | 148.9770 |
| 128 | Glu-Gly | C7H12N2O5 | 204.0746 | 205.0819 | M+H | 0.72 | 84.0444 | 130.0499 | 148.0604 |
| 129 | glu-ser | C8H14N2O6 | 234.0852 | 235.0925 | M+H | 0.71 | 118.0862 | 109.0763 | 60.0445 |
| 130 | Glutamine | C5H10N2O3 | 146.0691 | 147.0764 | M+ACN+H | 0.90 | 87.0440 | 146.1174 | 60.0809 |
| 131 | glu-thr | C9H16N2O6 | 248.1008 | 231.0977 | M+H-H2O | 0.75 | 70.0650 | 127.0320 | 186.1060 |
| 132 | glyceraldehyde 3-phosphate | C3H7O6P | 169.9983 | 171.0056 | M+H | 11.32 | 146.9613 | 128.9507 | 55.9346 |
| 133 | Glycyl-L-leucine | C8H16N2O3 | 188.1161 | 189.1234 | M+H | 3.15 | 86.0600 | 86.0964 | 143.1178 |
| 134 | Glycylproline | C7H12N2O3 | 172.0847 | 173.0920 | M+H | 0.71 | 70.0650 | 116.0705 | 127.0865 |
| 135 | Gly-l-pro | C7H12N2O3 | 172.0848 | 155.0815 | M+H-H2O | 0.91 | 70.0652 | 156.0420 | 155.0815 |
| 136 | GPK | C13H24N4O4 | 300.1796 | 301.1867 | M+H | 0.81 | 70.0652 | 127.0866 | 155.0814 |
| 137 | Guanazodine | C9H20N4 | 184.1689 | 185.1762 | M+H | 0.50 | 72.0808 | 69.0700 | 83.0855 |
| 138 | Guanine | C5H5N5O | 151.0494 | 152.0567 | M+H | 0.81 | 110.0349 | 128.0455 | 135.0301 |
| 139 | Guanine | C5H5N5O | 151.0494 | 152.0566 | M+H | 0.95 | 135.0300 |  |  |
| 140 | Guanosine | C10H13N5O5 | 283.0916 | 284.0989 | M+H | 2.88 | 152.0566 | 70.0652 | 153.0412 |
| 141 | Guvacine | C6H9NO2 | 127.0633 | 145.0971 | M+NH4 | 0.53 | 84.0808 | 72.0808 | 130.0863 |
| 142 | HC Blue No. 1 | C11H17N3O4 | 255.1218 | 256.1292 | M+H | 3.80 | 70.0652 | 257.1127 | 114.0550 |
| 143 | Histamine | C5H9N3 | 111.0797 | 112.0870 | M+H | 9.28 | 70.0652 | 69.0448 | 71.0605 |
| 144 | HO-dPEG8-OH | C16H34O9 | 370.2200 | 388.2538 | M+NH4 | 3.88 | 89.0597 | 133.0859 | 371.2269 |
| 145 | Homocitrulline | C7H15N3O3 | 189.1113 | 190.1186 | M+H | 0.77 | 84.0808 | 129.1021 | 130.0862 |
| 146 | Hydroxylysine | C6H14N2O3 | 162.1005 | 163.1078 | M+H | 0.53 | 103.0390 | 60.0810 | 85.0285 |
| 147 | Hypoxanthine | C5H4N4O | 136.0385 | 137.0458 | M+H | 0.91 | 119.0352 | 94.0400 | 110.0348 |
| 148 | Indane | C9H10 | 118.0783 | 119.0856 | M+H | 6.87 | 95.0491 | 91.0542 | 53.0389 |
| 149 | Indole-3-carboxaldehyde | C9H7NO | 145.0528 | 146.0601 | M+H | 3.53 | 122.0192 | 118.0651 | 124.0149 |
| 150 | Indoline | C8H9N | 78.0470 | 120.0808 | M+ACN+H | 3.09 | 103.0542 | 121.0841 | 93.0699 |
| 151 | Isobutyric acid | C4H8O2 | 106.0630 | 89.0597 | M+H-H2O | 4.77 | 72.0444 | 91.0542 | 72.0808 |
| 152 | L-(-)-Asparagine | C4H8N2O3 | 132.0535 | 133.0607 | M+H | 0.61 | 86.0600 | 68.0496 | 132.0655 |
| 153 | L(-)-Carnitine | C7H15NO3 | 161.1050 | 162.1122 | M+H | 0.59 | 103.0390 | 60.0809 | 85.0283 |
| 154 | L-(-)-Methionine | C5H11NO2S | 149.0511 | 150.0583 | M+H | 1.17 | 56.0497 | 104.0528 | 61.0108 |
| 155 | L(-)-Pipecolinic acid | C6H11NO2 | 129.0790 | 130.0863 | M+H | 0.53 | 84.0808 | 130.0862 | 85.0840 |
| 156 | L-(+)-Aspartic acid | C4H7NO4 | 133.0375 | 156.0268 | M+Na | 0.61 | 110.0713 | 155.0814 | 70.0653 |
| 157 | L-(+)-Citrulline | C6H13N3O3 | 175.0956 | 176.1030 | M+H | 0.65 | 70.0652 | 60.0558 | 116.0706 |
| 158 | lactide | C6H8O4 | 144.0423 | 145.0496 | M+H | 0.91 | 87.0440 | 146.1174 | 60.0809 |
| 159 | L-Ergothioneine | C9H15N3O2S | 229.0883 | 230.0956 | M+H | 0.82 | 60.0810 | 127.0320 | 186.1060 |
| 160 | L-Ergothioneine | C9H15N3O2S | 229.0883 | 230.0956 | M+H | 0.67 | 127.0320 |  |  |
| 161 | Leucylproline | C11H20N2O3 | 228.1475 | 229.1548 | M+H | 3.27 | 70.0652 | 229.1238 | 84.0444 |
| 162 | Leu-Gly-Pro | C13H23N3O4 | 285.1688 | 286.1761 | M+H | 3.56 | 70.0650 | 127.0870 | 155.0810 |
| 163 | Leu-pro | C11H20N2O3 | 228.1474 | 229.1547 | M+H | 0.91 | 100.0757 | 230.1861 | 72.0444 |
| 164 | L-Glutamic acid | C5H9NO4 | 147.0531 | 148.0603 | M+H | 0.64 | 84.0444 | 102.0549 | 130.0498 |
| 165 | L-Histidine | C6H9N3O2 | 155.0695 | 156.0768 | M+H | 0.60 | 110.0713 | 155.0813 | 130.0629 |
| 166 | linatine | C10H17N3O5 | 259.1170 | 260.1240 | M+H | 0.82 | 86.0600 | 189.0869 | 84.0444 |
| 167 | LNK | C16H31N5O5 | 188.1161 | 189.1234 | M+H | 0.81 | 84.0808 | 129.1022 | 130.0862 |
| 168 | sn-Glycero-3-phosphocholine | C8H20NO6P | 257.1028 | 258.1099 | M+H | 0.63 | 104.1070 | 125.0000 |  |
| 169 | LPE 17:1 | C22H44NO7P | 465.2854 | 466.2927 | M+H | 7.35 | 325.2735 | 62.0602 | 326.2777 |
| 170 | LPE 18:1 | C23H46NO7P | 479.3011 | 480.3082 | M+H | 7.65 | 339.2892 | 62.0602 | 340.2925 |
| 171 | L-Theanine | C7H14N2O3 | 174.1005 | 175.1077 | M+H | 1.90 | 72.0808 | 56.9650 | 57.0701 |
| 172 | Megastachine | C20H29NO3 | 331.2145 | 332.2216 | M+H | 7.34 | 91.0542 | 58.0654 | 333.3343 |
| 173 | Methyl cinnamate | C10H10O2 | 162.0681 | 163.0753 | M+H | 7.94 | 105.0335 | 163.0751 | 162.1274 |
| 174 | Methyl palmitate | C17H34O2 | 287.2822 | 288.2895 | M+H | 6.12 | 88.0757 | 289.2929 | 70.0652 |
| 175 | MFCD00025555 | C9H18N2O3 | 202.1318 | 203.1391 | M+H | 0.89 | 70.0652 | 144.0655 | 118.0862 |
| 176 | MFCD00037215 | C8H14N2O5 | 218.0902 | 260.1240 | M+ACN+H | 0.88 | 86.0600 | 189.0870 | 70.0652 |
| 177 | MFCD00037235 | C16H28N4O6 | 372.2007 | 373.2079 | M+H | 3.26 | 86.0600 | 260.1239 | 373.2077 |
| 178 | morpholine-4-carboximidamide hydrobromide | C10H10N2 | 158.0844 | 130.0975 | M+H | 0.91 | 84.0444 | 130.0499 | 84.0808 |
| 179 | MW_84.12489 | IS |  | 83.0146 | M+H | 0.51 | 71.0291 | 80.0343 | 68.5264 |
| 180 | Myristoyl Ethanolamide | C16H33NO2 | 271.2511 | 272.2582 | M+H | 5.98 | 254.2476 | 100.0757 | 86.0600 |
| 181 | N,N-Diethylaniline | C10H15N | 149.1205 | 150.1277 | M+H | 11.29 | 108.0682 | 150.1276 | 148.1119 |
| 182 | N,N-Dimethyldecylamine N-oxide | C12H27NO | 201.2093 | 202.2166 | M+H | 5.32 | 95.0492 | 57.0701 | 203.2202 |
| 183 | N,N-Dimethyldodecylamine N-oxide | C12H27NO | 229.2406 | 230.2479 | M+H | 5.93 | 57.0700 | 62.0600 | 71.0860 |
| 184 | N-{3-[(4-Acetamidobutyl)amino]propyl}acetamide | C11H23N3O2 | 229.1790 | 230.1863 | M+H | 2.05 | 100.0758 | 230.1862 | 229.1547 |
| 185 | N~2~-(1-Carboxyethyl)-N~5~-(diaminomethylene)ornithine | C9H18N4O4 | 246.2636 | 229.1295 | M+H-H2O | 0.73 | 70.0652 | 229.1158 | 132.0656 |
| 186 | N6-Acetyl-L-lysine | C8H16N2O3 | 188.1160 | 189.1233 | M+H | 0.67 | 84.0810 | 126.0913 | 86.0599 |
| 187 | N6-METHYLLYSINE | C7H16N2O2 | 160.1212 | 161.1283 | M+H | 0.51 | 84.0810 |  |  |
| 188 | N8-Acetylspermidine | C9H21N3O | 187.1685 | 188.1757 | M+H | 0.60 | 72.0808 | 100.0757 | 171.1491 |
| 189 | N-Acetylcadaverine | C7H16N2O | 144.1262 | 145.1335 | M+H | 1.23 | 84.9597 | 86.0964 | 95.0492 |
| 190 | N-Acetylornithine | C7H14N2O3 | 174.1005 | 175.1078 | M+H | 0.71 | 70.0652 | 60.0558 | 175.1191 |
| 191 | Nicotinamide | C6H6N2O | 122.0480 | 123.0553 | M+H | 0.81 | 80.0490 | 96.0440 |  |
| 192 | Nicotinamide | C6H6N2O | 122.0480 | 123.0553 | M+H | 0.75 | 80.0490 |  |  |
| 193 | Nicotinic acid | C6H5NO2 | 123.0320 | 124.0393 | M+H | 0.86 | 80.0494 | 96.0443 | 124.0585 |
| 194 | N-methylethanolamine phosphate | C3H10NO4P | 155.0347 | 156.0421 | M+H | 0.67 | 156.0420 | 110.0712 | 70.0652 |
| 195 | N-Nitrosoguvacoline | C7H10N2O3 | 170.0691 | 203.1027 | M+H+MeOH | 0.91 | 118.0862 | 203.1140 | 70.0652 |
| 196 | NP-015331 | C26H34O6 | 459.2616 | 460.2687 | M+H | 6.88 | 119.0855 | 135.0804 | 107.0855 |
| 197 | NP-019722 | C8H13NO4 | 204.1110 | 205.1182 | M+H | 0.75 | 84.0444 | 130.0499 | 148.0603 |
| 198 | NP-020014 | C15H26O3 | 276.1725 | 277.1798 | M+H | 7.93 | 235.1692 | 57.0702 | 278.1834 |
| 199 | NP-020454 | C6H13NO | 115.0997 | 116.1070 | M+H | 0.77 | 70.0652 | 116.0706 | 98.9842 |
| 200 | NP-021733 | C7H13NO2 | 160.1211 | 144.1019 | M+H+MeOH | 0.60 | 84.0810 | 56.0497 | 96.0523 |
| 201 | NP-022076 | C25H34O4 | 420.2249 | 421.2321 | M+H | 7.91 | 146.9818 | 321.1436 | 80.9479 |
| 202 | NPYR | C4H8N2O | 100.0637 | 101.0710 | M+H | 9.30 | 60.0446 | 59.0606 | 101.0710 |
| 203 | N-Undecanoylglycine | C13H25NO3 | 243.1834 | 226.1801 | M+H-H2O | 5.60 | 184.1696 | 226.1797 | 180.1746 |
| 204 | Oleamide | C18H35NO | 281.2717 | 282.2790 | M+H | 9.13 | 69.0699 | 83.0855 | 57.0701 |
| 205 | ophthalmic acid | C11H19N3O6 | 289.1273 | 290.1344 | M+H | 1.60 | 290.0859 | 58.0654 | 90.9767 |
| 206 | Oxadixyl | C14H18N2O4 | 278.1266 | 279.1340 | M+H | 3.33 | 120.0808 | 132.0656 | 136.0758 |
| 207 | Oxypurinol | C5H4N4O2 | 152.0335 | 153.0408 | M+H | 2.90 | 152.0566 | 153.0408 | 110.0349 |
| 208 | Palmitic Acid | C16H32O2 | 273.2665 | 274.2738 | M+H | 5.86 | 88.0757 | 70.0652 | 106.0862 |
| 209 | Pantothenic acid | C9H17NO5 | 219.1107 | 220.1180 | M+H | 3.25 | 90.0550 | 220.1177 | 202.1074 |
| 210 | PEG n11 | C22H46O12 | 502.2987 | 520.3327 | M+NH4 | 4.13 | 89.0597 | 133.0859 | 177.1121 |
| 211 | PEG n12 | C24H50O13 | 546.3247 | 564.3586 | M+NH4 | 4.20 | 89.0597 | 133.0859 | 177.1121 |
| 212 | PEG n13 | C26H54O14 | 590.3507 | 608.3848 | M+NH4 | 4.26 | 89.0597 | 133.0859 | 177.1121 |
| 213 | PEG n14 | C28H58O15 | 651.4033 | 652.4106 | M+H | 4.31 | 89.0597 | 133.0859 | 177.1122 |
| 214 | PEG n6 | C12H26O7 | 282.1678 | 283.1750 | M+H | 3.61 | 89.0597 | 133.0858 | 70.0652 |
| 215 | PEG n7 | C14H30O8 | 326.1939 | 327.2012 | M+H | 3.76 | 89.0597 | 70.0652 | 133.0859 |
| 216 | pentobarbital | C11H18N2O3 | 226.1318 | 227.1390 | M+H | 3.84 | 86.0964 | 86.0600 | 114.0549 |
| 217 | Phenethylamine | C8H11N | 121.0891 | 122.0964 | M+H | 11.29 | 88.0231 | 79.0179 | 56.9650 |
| 218 | Phenylacetaldehyde | C8H8O | 120.0575 | 121.0648 | M+H | 2.22 | 84.9597 | 122.0191 | 121.0647 |
| 219 | Phthalic anhydride | C8H4O3 | 148.0160 | 149.0233 | M+H | 8.13 | 121.0284 | 65.0387 | 150.0265 |
| 220 | Pilocarpine | C11H16N2O2 | 208.1212 | 226.1550 | M+NH4 | 2.82 | 125.0710 | 70.0652 | 84.0808 |
| 221 | Pinidine | C9H17N | 139.1361 | 140.1434 | M+H | 3.27 | 95.0492 | 53.0389 | 105.0448 |
| 222 | Piperidine | C5H11N | 85.0892 | 86.0965 | M+H | 0.52 | 56.9650 | 84.9597 | 69.0700 |
| 223 | porphobilinogen | C16H14N2O4 | 226.0954 | 227.1027 | M+H | 3.18 | 70.0650 | 141.0000 | 229.1230 |
| 224 | Zalcitabine | C9H13N3O3 | 211.0960 | 244.1292 | M+H+MeOH | 3.13 | 70.0650 | 127.0870 | 155.0810 |
| 225 | Pregabalin | C8H17NO2 | 159.1258 | 160.1331 | M+H | 0.72 | 53.0390 | 95.0491 | 105.0447 |
| 226 | pro-gln | C10H17N3O4 | 243.1218 | 244.1292 | M+H | 0.91 | 70.0652 | 127.0865 | 155.0814 |
| 227 | Proline-hydroxyproline | C10H16N2O4 | 228.1111 | 229.1184 | M+H | 3.55 | 70.0652 | 229.1547 | 116.0706 |
| 228 | Prolylleucine | C11H20N2O3 | 228.1473 | 229.1547 | M+H | 3.34 | 70.0650 | 86.0960 | 142.0860 |
| 229 | Pyrrolidine | C4H9N | 71.0735 | 72.0808 | M+H | 0.81 | 55.0540 | 57.0580 |  |
| 230 | RG5927903 | C13H22O2 | 210.1620 | 211.1692 | M+H | 6.16 | 70.0652 | 95.0491 | 212.0737 |
| 231 | RI2735000 | C9H14O2 | 154.0993 | 155.1066 | M+H | 5.07 | 72.9371 | 131.9742 | 113.9637 |
| 232 | sn-Glycero-3-phosphocholine 1:1 cadmium chloride adduct | C8H21NO6P | 257.1027 | 258.1099 | M+H | 0.59 | 104.1069 | 124.9997 | 258.1098 |
| 233 | sn-Glycero-3-phosphocholine 1:1 cadmium chloride adduct-2 | C8H21NO6P | 257.1027 | 258.1099 | M+H | 1.80 | 72.9370 | 86.0960 | 87.1000 |
| 234 | Spermidine | C7H19N3 | 145.1579 | 146.1651 | M+H | 0.50 | 72.0808 | 128.0192 | 105.0033 |
| 235 | Tetraacetylethylenediamine | C10H16N2O4 | 228.1111 | 229.1183 | M+H | 0.89 | 100.0756 | 230.1860 | 72.0444 |
| 236 | Threonine | C4H9NO3 | 119.0583 | 120.0655 | M+H | 0.63 | 74.0600 | 56.0497 | 102.0549 |
| 237 | Thymine | C5H6N2O2 | 126.0429 | 127.0502 | M+H | 2.64 | 84.9600 | 127.0501 | 110.0235 |
| 238 | toluidine | C7H9N | 107.0735 | 108.0808 | M+H | 11.29 | 68.0496 | 109.0763 | 67.0292 |
| 239 | Tranexamic acid | C8H15NO2 | 125.0840 | 158.1176 | M+H+MeOH | 0.91 | 95.0491 | 105.0448 | 53.0389 |
| 240 | L-(+)-Citrulline | C6H13N3O3 | 175.0956 | 176.1030 | M+H | 0.56 | 60.0560 | 70.0650 | 116.0710 |
| 241 | trans-3-Indoleacrylic acid-2 | C11H9NO2 | 187.0632 | 188.0707 | M+H | 3.48 | 118.0650 | 146.0600 |  |
| 242 | Trans-Cinnamic acid | C9H8O2 | 148.0524 | 149.0598 | M+H | 3.09 | 103.0542 | 79.0542 | 107.0491 |
| 243 | Tributyl phosphate | C12H27O4 P | 266.1646 | 267.1718 | M+H | 7.62 | 98.9841 | 155.0466 | 116.9947 |
| 244 | Trigonelline | C7H7NO2 | 137.0477 | 138.0549 | M+H | 0.91 | 94.0400 | 110.0350 | 119.0350 |
| 245 | Tris(2-butoxyethyl) phosphate | C18H39O7P | 398.2430 | 399.2503 | M+H | 7.92 | 57.0701 | 199.0729 | 101.0961 |
| 246 | U-48800 | C17H24Cl2N2O | 342.1224 | 343.1296 | M+H | 4.04 | 70.0652 | 229.1545 | 213.0668 |
| 247 | Uracil | C4H4N2O2 | 112.0272 | 113.0345 | M+H | 0.90 | 113.0597 | 69.0336 | 113.0345 |
| 248 | Valclavam | C14H23N3O6 | 329.1585 | 330.1657 | M+H | 3.18 | 70.0652 | 127.0865 | 155.0814 |
| 249 | Valpromide | C8H17NO | 143.1310 | 144.1383 | M+H | 5.61 | 122.0192 | 97.0076 | 88.0231 |
| 250 | Valyl-4-hydroxyproline | C10H18N2O4 | 230.1266 | 231.1339 | M+H | 0.91 | 100.0757 | 230.1861 | 72.0444 |
| 251 | Veronal | C8H12N2O3 | 184.0848 | 185.0921 | M+H | 3.51 | 84.9597 | 69.0700 | 83.0855 |
| 252 | vinyl sulfide | C4H6S | 86.0190 | 87.0264 | M+H | 0.91 | 56.9650 | 88.0756 | 76.5151 |
| 253 | Zalcitabine | C9H13N3O3 | 211.0960 | 212.1032 | M+H | 3.06 | 70.0650 | 95.0490 | 141.0000 |

The color filled in the table represents qualitative accuracy (Green: Accurate qualitative; Yellow: Relatively accurate qualitative; White: Fuzzy qualitative).

# Supplementary Table 4. Number of samples in the training set and validation set

| Strains | Model training | Number of samples in the training set | Number of samples in the validation set |
| --- | --- | --- | --- |
| *B.cereus*  *Cronobacter sakazakii*  *Escherichia coli*  *L.innocua*  *L.monocytohenes*  *Staphylococcus aureus* | 1408,14579,63301  1541,2991  25922,8739,8099,44102,44103  11288, 1969, 2016,2120,2941  2103-1,1834-2,1962-1,85-1  21593,29213 | 89  55  219  139  100  62 | 30  10  50  20  20  20 |


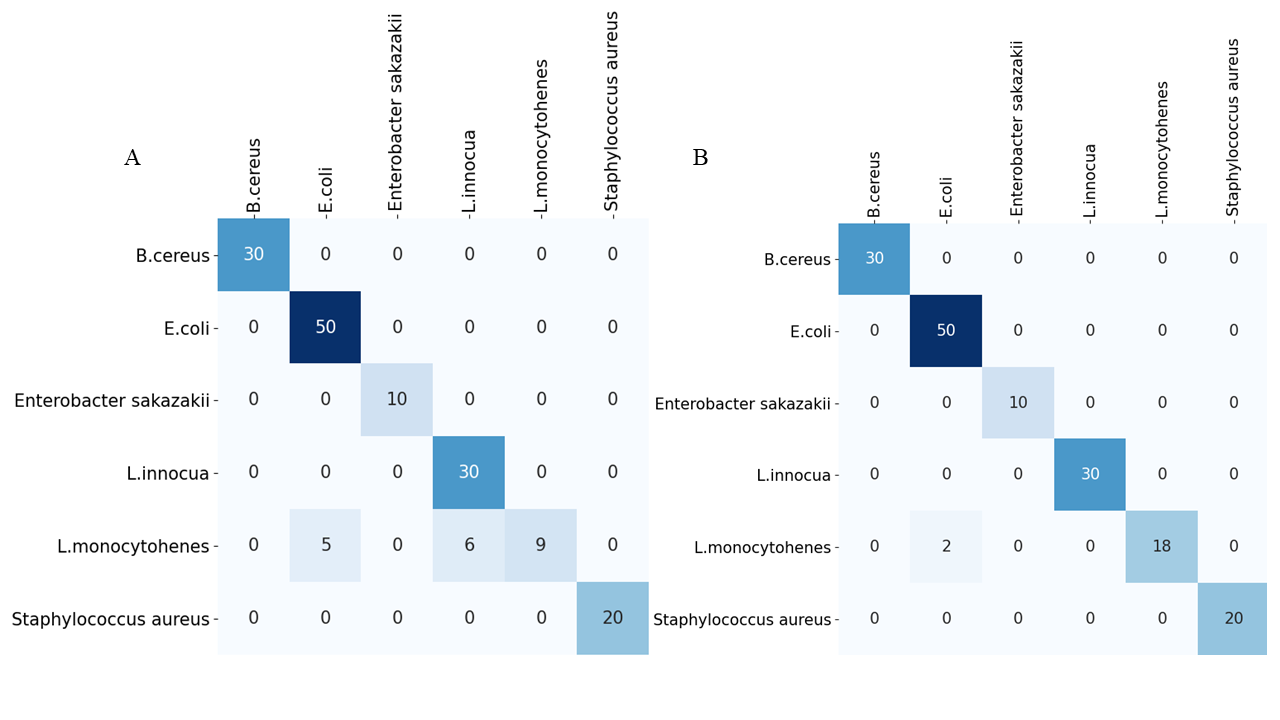
**Supplementary Figure 1**. The confusion matrix chart of the validated dataset predicted results. (A) The prediction accuracy of SVM was 93.13%. (B) The prediction accuracy of CNN was 98.75%
